# Social and Cultural Factors Influencing Health in Southern West Virginia: A Qualitative Study

**Published:** 2006-09-15

**Authors:** Cathy A Coyne, Cristina Demian-Popescu, Dana Friend

**Affiliations:** Department of Community Medicine, School of Medicine, West Virginia University; Mary Babb Randolph Cancer Center, West Virginia University, Morgantown, WVa; Department of Community Medicine, School of Medicine, West Virginia University, Morgantown, WVa

## Abstract

**Introduction:**

Social, cultural, and economic environments are associated with high rates of disease incidence and mortality in poor Appalachian regions of the United States. Although many historical studies suggest that aspects of Appalachian culture (e.g., fatalism, patriarchy) include values and beliefs that may put Appalachians at risk for poor health, other cultural aspects may be protective (e.g., strong social ties). Few recent studies have explored regional cultural issues qualitatively. The purpose of this study was to examine social and cultural factors that may be associated with health and illness in an Appalachian region.

**Methods:**

Ten focus groups were conducted in southern West Virginia and included five groups of men and five groups of women. Cultural norms associated with residents of rural Appalachia, such as faith, family values, and patriarchy, were examined.

**Results:**

Both men and women in the focus groups have a sense of place, strong family ties, and a strong spiritual belief or faith in God. Patriarchy as a cultural value was not a strong factor.

**Conclusion:**

There are limits to how qualitative data may be used, but findings from this study help increase understanding of the social and cultural environments of people living in rural Appalachia and how these environments may affect health.

## Introduction

For more than a century, Appalachia has been mythologized by journalists, scholars, and policy makers as an area apart from the rest of America in terms of geography, economy, and culture (1-4). Beginning in the 1870s, the local color literary movement characterized Appalachia as "a strange land with a peculiar people," isolated from mainstream America and its modernization (1,5). Characteristics attributed to Appalachians included fundamentalism, isolationism, familism, and homogeneity, and these characteristics soon "calcified into stereotypes" (2) that are still promulgated by the media and recognized by people living in this region.

Social scientists studying Appalachia have tried to confirm or dispel those myths and stereotypes and create a true picture of this diverse region. Most scholarly literature on Appalachian culture is dated and was published in the 1960s and 1970s (6-10). One of the first systematic empirical studies on Appalachian attitudes and beliefs was conducted in 1958 by Thomas Ford (6). Ford's survey was designed to measure fatalism, religiosity, individualism, and self-reliance — values traditionally ascribed to Appalachians. Ford's study refuted the existence of certain values (e.g., fatalism) and supported the existence of others (e.g., religiosity). Lewis and Billings noted that Ford's study influenced the work of social scientists and writers by identifying attributes that could be linked with Appalachian subcultures (2). Ford's study suggested that cultural attributes are dynamic and adapt to a changing social and economic environment, but we continue to find portrayals of Appalachia and its people in scholarly and popular literature that have not been substantiated by empirical research (11,12).

In this qualitative study, we examined cultural attributes and their association with health among a defined subgroup of adults living in Appalachia. We chose to use focus group methodology because it allowed us to explore issues in a social environment and to gather qualitative data. In this report, we included quotes from participants to illustrate social and cultural themes that emerged from the focus groups and considered how these social and cultural values may affect health.

## Methods

Ten focus groups were conducted; five were composed of men, and five were composed of women. Groups were separated by sex to enable candid discussions about gender roles and family decision making. Purposive sampling (13), a method to select participants who will provide information of central importance to the study, was used to recruit participants from five southern West Virginia counties. Participants met the following eligibility criteria: 1) are aged 35 years or older, 2) have lived most of their lives in West Virginia, and 3) have parents and grandparents born and reared in the state. These eligibility criteria were used to recruit participants who were likely to reflect the Appalachian culture found in the southern part of the state. Participants came from Boone, Fayette, McDowell, Mercer, and Raleigh counties — areas that represent the southern coalfields of the state. Although populations in these counties are not homogeneous, they are more similar to one another than they are to other West Virginia populations in terms of income, race, ethnicity, and occupation.

This study was approved by the West Virginia University School of Medicine's Institutional Review Board. Participants were recruited by community organizations and agencies (e.g., local American Cancer Society, local cooperative extension, rural clinics, service organizations). Focus groups were held at sites and times that were convenient and comfortable for participants.

The research team developed a questionnaire based on input from a community advisory committee to guide focus group discussions. The advisory committee included community members from southern West Virginia and other regions of the state. Several individuals were members of cancer coalitions affiliated with the Appalachia Cancer Network, a special populations network funded by the National Cancer Institute (14). Discussions included queries about values involving self-identity and individual perceptions about patriarchy, religious faith, gender roles, and fatalism — values that are frequently associated with this region in literature about Appalachia.

Participants gave permission for all discussions to be audiotaped, and all sessions except one were facilitated by one of the authors. Audiotapes were transcribed and analyzed (Ethnograph 5.08, Qualis Research, Colorado Springs, Colo). We used content analysis techniques (i.e., examined quotations for common ideas or themes) to identify and code transcript sections based on preestablished social and cultural value categories. Codes, such as *value* or *belief*, were then assigned to category sets of preliminary main themes, such as *religiosity*, or subthemes, such as* religious practices*.

When participants arrived at the focus group site, they were asked to review an informed consent statement written at a sixth-grade reading level. Before participants were asked to sign the statement, the facilitator read it aloud to each group. Each focus group lasted approximately 2 hours. Each participant received $50 at the end of the focus group discussion as compensation for time contributed.

## Results

There were 61 participants, 31 women and 30 men, in the study. Sixteen participants were African American, and the remaining 45 were white (Table).

### West Virginian identity

Participants in all groups attributed the following characteristics to West Virginians: 1) kind, 2) outgoing, 3) openhearted, and 4) helpful. The most frequent responses describing the strengths of West Virginians were 1) spiritual beliefs or faith in God and 2) family values. Other characteristics considered as strengths by most participants included 1) good moral values, 2) a sense of community, 3) commitment and dedication to work, 4) mutual respect, 5) hospitality, and 6) pride. Participants reported that people in the region are proud to live in West Virginia and feel offended when others consider them indigent because of where they live.

Study participants characterized West Virginians as hard-working and as having a willingness and desire to work. Terms used to explain the value placed on work included 1) a strong work ethic, 2) loyalty, 3) dependability, 4) trustworthiness, and 5) dedication. Participants reported that West Virginians appreciate the values work instills and that people respect all types of work and workers. One respondent stated, "I think women in this area are strong. I think they had to be strong because of the way they have been brought up — the hard work involved. I think they had to maintain being the strength for their family, for their husband and children."

A deep sense of place emerged from all focus group discussions and was exemplified by the attachment participants felt to "their mountains" and the sense of belonging they verbalized. Most respondents stated that they could not understand why anyone would ever want to leave the state. A few who left for a short time to work elsewhere reported that they could not wait to get back. They missed the mountains as the following quote exemplifies:

You go out here and get back in these mountains and just look at that scenery. . . . Makes you wonder why people want to leave and everything like this. It's beautiful in the state. You know, it's God's creation, and our mountains are ours — and we love them.

### Appalachian identity

Initially participants objected to being referred to as Appalachian. None identified with the stereotypical characteristics of Appalachians (e.g., uneducated, poor, lacking intelligence). Participants stated: 1) "When I hear *Appalachia*,. . . I think of extreme poverty; we are poverty-laden people, but this [the stereotype of Appalachia] is extreme poverty"; 2) "Somebody with no shoes on, living in a shack. . . . Back up in the mountain, up in the hills. No running water. No TV. Probably no refrigerator. With no food. That's what I think of. Yeah. And we're not like* that.*"**

When the word* Appalachian* was used as a geographic descriptor, the term was regarded as an acceptable label because participants recognized they indeed did live in the Appalachian region. Positive attributes of people living in Appalachia voiced by participants included being 1) friendly, 2) God fearing, 3) proud, 4) law abiding, 5) hard working, 6) clannish, and 7) reluctant to share family problems. These characteristics reflected how participants felt about themselves and others in their communities.

Numerous personal stories emerged when Appalachian and West Virginian stereotypes were discussed. Most participants had an experience to share in which they or a family member was treated with disrespect because of where they lived. One man in his 50s shared the following story:

When I went in the military, I was going through the. . . line where they [were] distributing equipment. And I walked up, and the guy asked me where I was from. And I said "I'm from West Virginia." And he said, "What part?" I said, "I'm from southern West Virginia." And he tossed me my boots and says, "Your first pair of boots." I just wanted to look at him, and you know it kind of touched a feeling in you. I said, "Go on and believe it, but that's not my first pair of boots." And that was like we're running around here barefoot.

Another participant shared frustration about how people react to the way southern West Virginians speak: "If you open your mouth, and you have a West Virginia accent or a Kentucky accent, when you open your mouth, your IQ immediately drops thirty points — they assume that you're stupid."

Participants had a positive reaction to the term *hillbilly*; this term resonated more with them than the term* Appalachian*, as illustrated by the following quote: " . . . well my view is I'm a hillbilly, and I'm proud of it. But my idea of a hillbilly is not the world's perception of a hillbilly. To me, my perception of a hillbilly is somebody who loves the mountains, who loves their family, who loves their home, who loves this way of life."

Participants blamed the media's sensationalism for the establishment and perpetuation of negative Appalachian stereotypes. They felt stereotypes are conceived by people with little knowledge of the area's geography and culture. Participants felt that the derogatory meaning of stereotypes and the fact that negative images of West Virginia are not balanced with positive ones are what hurt people. Participants shared the following statements:

I don't feel like we are given the respect from the rest of the country that we should be given. Because I think what they look at in saying hillbilly is something derogatory, you know, and that we are ignorant people. And we may have a southern speech, but we are not ignorant people. We are smart people, and we have good values. . . . I don't feel like it [that the way we talk] should be something to make fun of us.

We haven't been individualized; we have been categorized.

### Egalitarian social organization 

The traditional family structure that includes a wife who stays home to rear children and a husband who is head of the household and provides financial support has changed in many Appalachian families. Both parents typically work outside the home and share decision making. Most men in the focus groups thought older generations lived in a more patriarchal society in which men held decision-making roles and women were caregivers and disciplinarians. Some women in the groups reported erosion of patriarchal roles in their communities that has resulted in women being expected to be decision makers in what were traditionally men's roles. According to participants, role changes sometimes occurred because men worked long hours and passed their responsibilities on to women.

Discussions among participants suggest that people believe the way patriarchal decision making in West Virginian families is portrayed is more a stereotype than reality. Most participants stated that family decisions are made jointly by husband and wife: "My wife and I, we discuss things." When decisions are the responsibility of one person over another, the basis for who made the final decision is related to who earned the family income and who had the most knowledge or authority, regardless of sex.

Feedback from participants indicated that decisions about when and where to seek medical care are made mutually by the man and woman in a family. When a family member has been diagnosed with a serious medical condition, treatment options are discussed among all family members before making a decision. When there is trust in the family physician, he or she generally makes the final decision about medical treatment.

Decisions about food preferences and what foods to prepare are no longer made based on the preferences of the man in the family or other family members. Respondents indicated a woman is not expected to prepare food based on individual choices made by family members; it is up to the woman to decide what to prepare. In addition, there are no longer strict rules about who does the cooking, what is prepared, and when to prepare food.

### Religion and faith in God 

Religion, family cohesion, friendship, health, and integrity are important values according to participants. They also indicated faith in God is important when a person needs support. Faith appears to be a traditional value that has been carefully passed down from one generation to another. Many participants believe that deep religious faith has its origins in the early years when West Virginia was settled. Life was hard, and people sought relief in religious faith. Spiritual beliefs offered emotional and spiritual support, and churches served as a bonding element in communities. Some participants identified with being in "the Bible belt" and stated they are more religious than people living in other parts of West Virginia. Two quotes illustrate this point:

The southern part of West Virginia is the Bible belt, you know. We have strong beliefs, and it's not beliefs you just pick up. It's beliefs that have been passed down from generation to generation. Always. A strong sense of family.

. . . if you would ask me what's the main purpose in my life, I would tell you right straight — it's serving God. . . . Because I feel like without serving God, I am nothing. . . . And that's the majority of. . . everybody."

Both men and women living in Appalachia tend to have a strong faith in God. Some participants pointed out differences between the sexes in the practice of worshiping God. Women go to church more often than men, they said, and men prefer to spend time at home or doing things in which they are interested (e.g., fishing with their children, watching NASCAR racing).

### Family cohesion 

Family cohesion is an important value passed down from generation to generation. Some participants reported family attachment is stronger than attachment to religion, especially among men. Participants stated that the role of a family is to provide care and education to children, and the power of example is critical in this process. Family gives people a sense of togetherness and is a resource for problem solving. Many families rely on grandparents or other relatives to help them with child rearing or for support in times of crisis. Participants noted that large, closely knit families are representative of  communities in West Virginia and are "almost a circle that can't be broken." Most participants stated that living close to family is important to them.

Participants reported that family members usually deal with family problems internally and that sometimes second-degree relatives, such as aunts, uncles, or grandparents, are included in the process. Although family problems are considered private and not to be shared with the community, an exception is sometimes made by involving the church. However, the preference is for family members to deal with problems, such as family violence or neglect, rather than to seek help from social services. Agencies are regarded as replacements for parental authority or family decision making. Families often do not allow their children to know about problems the families face. When they do know about problems, children are told not to discuss them beyond the family circle. This attitude is characterized by some of participants as manifestation of pride and is a value reported by many participants.

### Health-related issues 

According to most participants, health beliefs are strongly related to religious beliefs and practices among people living in southern West Virginia. Participants stated those living in the region tend to have low levels of medical knowledge, and they believe this lack of knowledge may be the reason few people in southern West Virginia discuss physical or mental health issues with those outside the family. In addition, participants recognized that many health problems affecting people in their region were related to health behaviors. "We have a lot of health problems, a lot of sick people, and one thing they say is we have a lot of obese people in this area. We like to eat, and we don't really exercise."

Depending on personal experiences with the medical system, participants reported that some southern West Virginians distrust physicians or question the quality of care they receive. Some participants report that seeking help from a medical institution or provider is regarded as a last resort among people in the region. There is distrust of specialists because people fear they will prescribe medication that could cause addiction, and there is concern that family problems will become public knowledge. Some participants did admit, however, that they knew of situations when specialized care was sought and resulted in a positive outcome.

Some participants expressed concern about a lack of American-born physicians in their geographic area and seemed disgruntled about having to see a foreign-born physician for medical care. Cultural differences between foreign-born physicians and the local population were reported and cited as a barrier to establishing a trusting relationship between patient and provider. Participants also expressed concern about the high turnover of medical providers in the region and stated this turnover makes it difficult to build trust.

Religious beliefs and faith in God are important resources when people in this area of West Virginia face sickness and seek healing. Participants reported that, for some West Virginians, divine help for healing seems to be enough but that others consider both faith and medical care when seeking a solution to health problems. This attitude is shown in the following statement: "I believe in prayer, but I believe [God] gave doctors knowledge, also." Many people in the region do rely on medical care for their health, but participants noted that care is often sought later rather than earlier. Participants stated that people in the region regard disease and accidents, like other hardships, as always a part of their lives.

Finding affordable health care was identified by some participants as a barrier. Many families do not have health insurance or have insurance with restrictions that inhibit access to care. Long distances to medical facilities are another barrier to those seeking care.

## Discussion

The findings from this qualitative study suggest that not all cultural attributes traditionally ascribed to Appalachians are found among people living in this region. Cultural attributes of people living in southern West Virginia that emerged from the study included a steadfast faith in God, strong family ties, and pride. Other cultural factors attributed to stereotypical Appalachians were not found. Literature on Appalachian culture and values often identifies this region as having a patriarchal social order in which the man is head of the household (10). Participants in this study rejected the patriarchal characterization and provided rationale for why this concept no longer characterizes their communities. Economic and social demands have created a more egalitarian society in which decisions are mutually made by husband and wife. This view is consistent with what Oberhauser describes as the effects of economic restructuring on relations between the sexes (15). She noted that the loss of male-dominated jobs because of mine closures and mechanization of the mining industry have resulted in changes in how income is generated and, subsequently, in how social relations are structured. Because focus groups were conducted in a traditional coal-mining region of West Virginia, most participants came from coal-mining families, and some were miners themselves. Focus group participants acknowledged that patriarchy was the model for previous generations but reported that even in earlier generations women exerted covert influence in decision making that was recognized by the children if not by the husband. Male dominance may have been a traditional value in the past but was not pervasive in all facets of life.

Fatalism is another stereotypical Appalachian cultural characteristic that did not emerge as a strong factor during focus group discussions. Although faith in God was found to be an important factor in the lives of most group members, participants did not find faith to be a barrier to obtaining health care. They did not voice the belief that health problems are determined by fate but recognized that lack of exercise and unhealthy diets are associated with poor health. Participants stated that most people in the region do seek health care when it is needed. People turn to prayer when illness affects the family, but participants stated that prayer is not usually used in lieu of seeking medical care from a health provider. Limited health-seeking behaviors are often influenced by lack of knowledge about health care rather than simple reliance on faith.

A powerful sense of place emerged in all focus groups. Sense of place has been defined as attachment to or identity with a specific community (16) that provides a collective meaning to a group. Place identity was exemplified by focus group participants through their connection with the term *hillbilly* and their enthusiastic description of the mountains as *home*. Place attachment, place identity, and place dependence all surfaced to varying degrees in statements made by participants about West Virginia.

Inclusion of participants from only a small region of Appalachia can be viewed both as a strength and a limitation of the study. We cannot use these findings to characterize other parts of Appalachia; however, the small regional approach we used enabled us to explore potential cultural factors within a relatively homogeneous region. Findings across all 10 groups for both men and women were consistent. We anticipated there would be some differences in perceptions of roles for the sexes and that these would emerge in groups that were separated by sex. Groups, however, were consistent in their opinions of roles of the sexes as well as on issues such as faith in God and a sense of place.

Other studies have demonstrated that culture can have profound influence on health behaviors. It is important for scientists and medical practitioners working with Appalachian communities to understand the culture of the area. It is not sufficient to rely on dated scholarly or popular literature that portray stereotyped Appalachian characteristics and culture or on overgeneralized attitudes and beliefs that may apply only to a subgroup of people. As many social scientists and geographers have begun to realize, Appalachia is a region of diverse people and resources. Physicians, health educators, policy makers, and behavioral scientists need to recognize this diversity so that health communities can work together to address the health needs of Appalachian America.

## Figures and Tables

**Table:** Sex and Race of Focus Group Participants by County, West Virginia, 2002

**County **	**Participants (N = 61)**			

	**Men**		**Women**	

	**African American (n = 11)**	**White (n = 19)**	**African American (n = 5)**	**White (n = 26)**
Boone	0	6	0	7
Fayette	4	3	3	6
McDowell	3	2	2	3
Mercer	1	3	0	5
Raleigh	3	5	0	5
